# Development and validation of a composed canine simulator for advanced veterinary laparoscopic training

**DOI:** 10.3389/fvets.2022.936144

**Published:** 2022-10-05

**Authors:** Carlos A. Oviedo-Peñata, Gloria E. Giraldo Mejía, Carlos Humberto Riaño-Benavides, Juan G. Maldonado-Estrada, Juan D. Lemos Duque

**Affiliations:** ^1^Tropical Animal Production Research Group, Faculty of Veterinary Medicine and Zootechny, University of Cordoba, Monteria, Colombia; ^2^OHVRI-Research Group, Faculty of Agrarian Sciences, College of Veterinary Medicine, University of Antioquia, Medellín, Colombia; ^3^Bioinstrumentation and Clinical Engineering Research Group-GIBIC, Department of Bioengineering, Faculty of Engineering, Universidad de Antioquia, Medellín, Colombia

**Keywords:** gastropexy, veterinary education, laparoscopic training, laparoscopy, minimally invasive surgical procedures, validation study, veterinary surgery, simulation training

## Abstract

The development of innovative simulation models for veterinary laparoscopic surgery training is a priority today. This study aimed to describe a didactic simulation tool for the training of total laparoscopic gastropexy (TLG) with intracorporeal sutures in dogs. CALMA Veterinary Lap-trainer composite simulator (CLVTS) was developed from a plaster cast of 2 Great Dane canines mimicking the space and the correct position to carry out a TLG. After video instruction, 16 veterinarians with different degrees of experience in minimally invasive surgery (Experts, *n* = 6 and intermediates, *n* = 10) evaluated four sequential simulating TLG with intracorporeal suturing in the CLVTS. Subsequently, they completed an anonymous questionnaire analyzing the realism, usefulness, and educational quality of the simulator. The CLVTS showed a good preliminary acceptance (4.7/5) in terms of the usefulness and adequacy of the exercises that, in the participants' opinion, are appropriate and are related to the difficulty of the TLG. In addition, both experienced and intermediate surgeons gave high marks (4.5/5) to the feeling of realism, design, and practicality. There were no significant differences between the responses of the two groups. The results suggest that the CVLTS has both face and content validity. Where it can be practiced in a structured environment for the development of a total laparoscopic gastropexy with intracorporeal suture and without compromising patient safety, but still has some limitations of the scope of the study. Further studies are needed to establish the ability to assess or measure technical skills, including the degree of transferability to the actual surgical environment.

## Introduction

Gastric dilatation and volvulus (GDV) have been cataloged as life-threatening syndrome in large-breed, deep-chested dogs. The stomach rotates on its axis, dilating and increasing intragastric pressure, leading to portal hypertension, systemic hypotension, and cardiogenic shock (1). To form serosa-to-serosa adhesions permanently fixing the stomach to the ventral abdominal wall (2), gastropexy has been used since 1979 to treat and prevent recurrence of GDV syndrome, reducing the rate of disease presentation to less than 5% in dogs subjected to gastropexy (3). There are multiple prophylactic gastropexy techniques such as endoscopic-assisted gastropexy (4), laparoscopic-assisted gastropexy (5), and TLG with intracorporeal sutures (6, 7). Laparoscopic techniques continue to gain popularity in dogs of at-risk breeds or those undergoing splenectomy for torsion or other splenic pathology because it is the least invasive alternative; however, it requires special equipment and significant surgical experience for the surgeons to perform it (3, 6). Even these surgical techniques have been reported as a treatment method in patients with GDV syndrome (8, 9).

The ability to perform intracorporeal suturing allows minimally invasive surgeons to be more versatile when performing different procedures with a purely laparoscopic approach rather than laparoscopically assisted (10). Surgeons prefer the laparoscopic-assisted gastropexy technique because laparoscopic total gastropexy with intracorporeal suturing requires a sufficient level of experience in intracorporeal suturing to minimize operative time and surgical risk since it is a complex technique and is commonly associated with prolonged surgical times (11, 12). The difficulties of pure laparoscopic gastropexy not only rely upon performing the intracorporeal suture but also on executing the suture knots required to bring the stomach closer to the abdominal wall. This limitation was overwhelmed by the introduction of barbed sutures (10).

Laparoscopic suturing skills do not automatically transfer from suturing skills learned in open surgery (13). Mattar et al. conducted a national survey to assess the skills of general surgery graduates entering accredited surgical subspecialty fellowships in North America. The author identified significant shortcomings in advanced laparoscopic skills, such as suturing and knot tying, as 56.2% of surgeons in training could not perform laparoscopic suturing while complying with the mandatory Fundamentals of Laparoscopic Surgery (FLS) certification (14). Other considerations that make relevant laparoscopic gastropexy training are the possible complications such as gastric perforation, splenic laceration, and cardiovascular instability due to abdominal insufflation or gas embolism. It is, therefore, essential for the laparoscopic surgeon to have rigorous training and experience to avoid intraoperative complications and obtain low failure rates (3).

The importance of simulation training and the creation of devices for training veterinary laparoscopic surgery is their capability to reproduce the difficulties inherent to minimally invasive techniques (15). Because the acquisition of skills necessary to perform laparoscopic surgery is not directly transferable from open surgery experience, the laparoscopic surgeon trainee must adapt to the loss of depth perception, fulcrum effect, and limited tactile feedback (16, 17) compared to conventional surgery. Because it is essential to measure the accuracy of a training and assessment method or instrument to measure specific skills (18), validating simulation devices and their training curriculum must be conducted before establishing the purpose used in teaching centers. Three frameworks are currently described for the validation of surgical skills: the classical validity framework (face, content, criterion-construct and predictive-, and concurrent) (18, 19), the framework for validation inference proposed by Kane (19), and a unified model of different validity sources called the modern Messick validity framework that has become the gold standard when evaluating validity evidence for performance assessments (20, 21).

Although there are validated simulators that allow the acquisition of basic skills in veterinary medicine, there are few low-cost, high-fidelity simulators (15, 17, 22). Likewise, there is a lack of studies on the construction of simulators and their respective micro-curricula for a particular surgical skill or advanced surgical technique (17, 23). For both basic and advanced training in laparoscopic surgery, the main limitation in training veterinary surgeons is that it requires surgery simulators designed for human use. The size of operational space is a significant limitation because they have standard sizes contrary to the variety of patient sizes in veterinary medicine. Another limitation is the position of the working ports to meet the triangulation principle and the natural position of canine patients on the surgical table. These factors influence training with simulators designed for human patient training (24). The present study aimed to describe the development of a simulation model and training plan for TLG with intracorporeal suturing in dogs and evaluate the face and content validity as a method of teaching the technique. We hypothesized that the didactic simulation medium provides a safe, controlled, and standardized environment that does not compromise patient safety for TLG training.

## Materials and methods

### Study design

An analytical, experimental study.

### Model development

**Figure 1:**
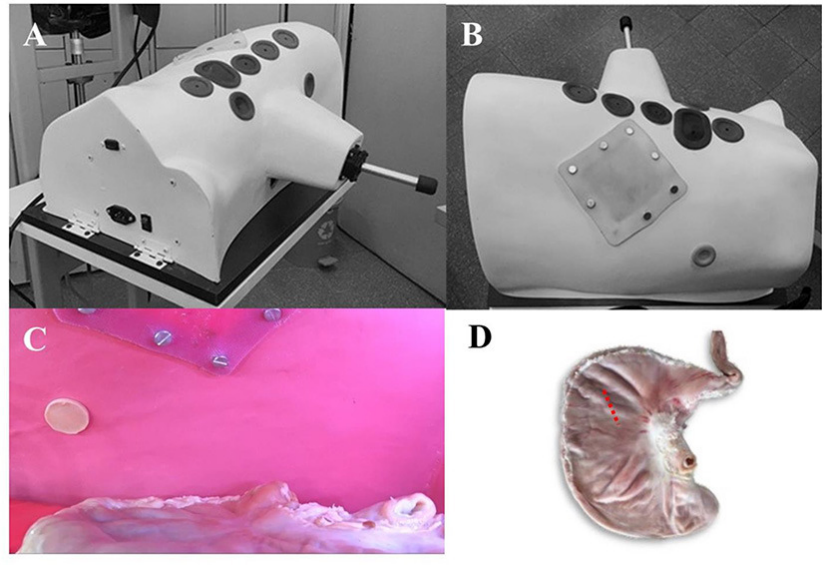
Photographs of the simulation model for performing pure laparoscopic gastropexy in canines (CALMA Veterinary Lap-trainer Simulator – CVLTS) **(A,B)** external appearance of the simulator, showing the arrangement of the linear ports and the simulated silicone abdominal wall. **(A)** Ventro-lateral view of the simulator showing the arrangement of the ports. For the TLG, 1 (subxiphoid) and 4 (paraumbilical) are used. **(B)** Dorsal view of the simulator where the silicone patch that mimics the abdominal wall can be seen where the percutaneous needle is passed and the intracorporeal suture is performed internally. **(C)** Internal view of the simulator. The stomach is shown ventrally in position to be anchored. **(D)** Position of the stomach during training.

For the creation and development of the CVLTS (Figures 1A,B), readily available and inexpensive materials were used. Considering the incidence of the disease, two male Great Dane Breed dogs that required prophylactic total laparoscopic gastropexy were taken. After performing peripheral catheterization in the MAD cephalic vein (catheter # 18) and with maintenance fluids at 3 ml/kg/hr, premedication with dexmedetomidine 2 ug/kg was applied. IV, Dipyrone 25 mg/kg IV and Cephalothin 25 mg/kg. IV. Induction, before pre-oxygenation (5 min at 5 Lt/min of Oxygen), was performed with Ketamine 0.6 mg/kg. IV, Fentanyl 1 ug/kg. IV and Propofol 2 mg/kg. IV. After orotracheal intubation (tube # 10.5), maintenance was performed with 2% isoflurane (oxygen flow 1 Lt/min).

Preparation of patients to be cast: Patients were positioned dorsoventral with an angulation of 25° to the left and aseptically prepared for routine intracorporeal suture pure laparoscopic gastropexy procedure. At the end of the procedure, the intra-abdominal pressure was controlled at 7 mmHg through a Veress needle placed in the infra umbilical region. The preparation of the negative mold is the one that will reproduce the shape of the canine torso “from inside”, with the exact shape of the torso and the angulation of a patient undergoing LGT, for which plaster bandage strips were used (VM Vital -Medic 6” × 5 yards) fast-drying to a 4 mm thickness on the abdominal cavity, trying to place them in all possible directions forming a resistant and well-adhered interlacing. To prevent the plaster from adhering to the area's skin to be replicated, the trunk of the patients was isolated with Vinipel paper (Tami Vinipel Wrap 200 × 305 mm). The cast made of plaster bandages was removed with care not to damage it and to avoid any harm to the patients. Once withdrawn, the patients recovered, and Meloxicam 0.1 mg/kg IV was administered. All patients had an uneventful recovery from anesthesia. The positive mold was created from the negative mold by covering it inside with fiberglass and epoxy resin in a mannequin-making workshop. The CVLTS has the following characteristics: It is an inanimate box made that simulates the ventral thoracoabdominal region of a large breed canine from the entrance of the pelvis to the mid-thoracic region with a slight inclination to the left. The dimensions are 46 cm long and 30 cm wide at the base. Its anterior border is 27 cm high up to the thorax (sternal region) and 14.6 cm up to the highest point of the pelvis (penis), creating a working cavity of approximately 15.000 cm^3^. The floor is made of wood, on which plastic-coated expanded polyethylene support is placed to hold the pig stomach (Figures 1C,D). The mold was designed with five linear silicone holes to allow the placement of conventional laparoscopic sleeves, four circular holes on the mid-ventral line (alba line), and one oval hole directed 2 cm toward the right side to improve triangulation for the pure laparoscopic gastropexy procedure. It also has two inferior circular silicone ports on both camera sides. In the internal part of the model corresponding to the location of the costal arch, a 12 × 12cm silicone suture pad simulating the abdominal wall was placed over the right mid-lateral region. This modification allows for comfortable and practical insertion angles that allow inert materials and *ex vivo* tissue for basic and advanced laparoscopic skills training. It has LED lighting and a separate port for the internal digital camera (Sony Action Cam FDR-X3000 4K Full HD 1080p at 120 fps adjustable) with different degrees of movement and even zoom in and out like a conventional rigid lens. The simulator was used on a mobile metal base with manually adjustable height from 60 to 120 cm, which adjusts to a 40” full HD image monitor TV with an adjustable arm in distinct positions.

### Evaluation of CVLTS

At convenience, a group of sixteen veterinary surgeons practicing minimally invasive surgery (MIS) with varying degrees of experience was divided into two groups according to the number of procedures and years of experience in MIS. Advanced-experienced group (Group A, *n* = 6) was composed of six expert veterinarians who perform multiple laparoscopic and thoracoscopy procedures and who routinely perform the technique of total laparoscopic gastropexy (TLG) with intracorporeal suture in dogs. Non-experienced group (Group B, *n* = 10), veterinarian surgeons who perform multiple minimally invasive surgical procedures, including laparoscopically assisted gastropexy, but not TLG.

All participants could perform four sequential exercises required to complete the TGL using intracorporeal on the CVLTS and analyze its didactic usefulness. A survey recorded demographic information, MIS years of experience, and previous simulator. All participants completed an anonymous 5-point Likert-type satisfaction survey to evaluate the realism of the model and its usefulness for training the TLG technique. All participants were required to complete the following basic steps of TLG using intracorporeal suturing, which was performed *ex vivo* using fresh postmortem pig stomach and surgical-grade laparoscopic instruments:

#### Anchoring suture

**Figure 2:**
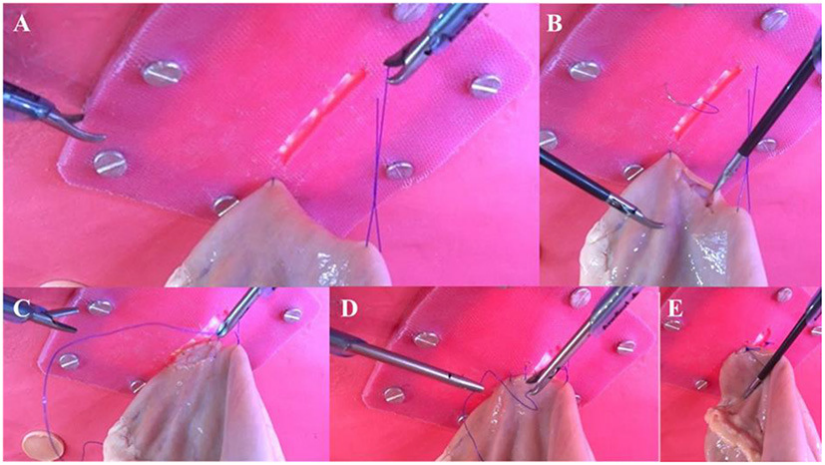
Internal images of the tasks of anchoring **(A)**, cutting **(B)** and suturing of the lateral **(C)** and medial **(D)** sides of the total laparoscopic gastropexy on the simulator. Laparoscopic gastropexy completed **(E)**.

Anchoring exercise (suture approximation of the stomach to the abdominal wall), which was performed with two non-absorbable monofilament polyamide sutures (nylon) N° 2–0, 75 cm long, with a 3/8 circle needle of 35 mm cutting tip, which was passed percutaneously (siliconized skin) at the planned gastropexy site (2–3 cm caudal to the last rib and 5–8 cm lateral to the midline). The needle was grasped with a laparoscopic needle holder inside the cavity, and a full-thickness deep bite was taken through the antrum of the pig stomach (*ex vivo* tissue). The suture was passed through the abdominal wall adjacent to its anterior entry point. On the outside of the abdominal wall, the suture ends were held in place with a Kelly clamp. This maneuver was repeated at 5–6 cm between both stitches to temporarily anchor the stomach to the abdominal wall during incision and suture. In addition, this second anchor point allows the stomach tension to be reduced during the suturing maneuver (Figure 2A).

#### Cutting exercise

It consisted of the ~4–5 cm serous-muscular layer of the stomach incision, using laparoscopic Metzenbaum scissors. The incision of the serosal-muscular layer of the abdominal wall was previously established in the siliconized cast (which simulates the abdominal wall). These incisions will be adjacent in an orientation parallel to the last stitch (Figure 2B).

#### Suturing exercises

Suturing exercises (suturing of the anterior or lateral face and suturing of the posterior or medial face) with monofilament non-absorbable suture No. 2–0, 19 cm long, with a round tip $\frac{1}{2}$ circle needle of 25 mm in a simple continuous pattern. The introduction of the needle to the simulator was performed percutaneously through the silicone skin adjacent to the gastropexy site. First, the lateral (anterior) wall of the seromuscular layer of the pig antrum was sutured to the lateral edge of the previously made siliconized skin incision (Figure 2C). Once the previous exercise was completed, the second piece of suture was introduced, and the medial (caudal) margins were sutured to complete the gastropexy (Figures 2D,E). After completion of the suture, the excess material was removed, and the procedure was completed.

### Visual analog scale assessment

Previous experience of participants was evaluated using a comparable visual scale that measured simulator use (0–100 mm): 0 mm indicated that they had never done simulator training at CMI, and 50 mm indicated that they had occasionally used simulators. In short, representative courses or commercial samples. Finally, 100 mm indicated that they had received rigorous training, under a reasonable and structured curriculum plan, with weekly repetitions for weeks or months. The exercises were conducted under one of the authors (CO-P). Additionally, a movement tracker was placed on the back of the hands of each participant for analysis in subsequent studies, and each procedure was recorded.

### Satisfaction survey

The questionnaire for evaluating the Satisfaction survey is presented in Table 1. We constructed a compact survey including a set of questions associated with the content and appearance of the simulator. The first seven questions evaluated the content of the simulator. The rest of the questions evaluated the appearance of the simulator (Table 1).

**Table 1:** Total distribution of responses and percentage of responses by group.

**Survey items**	**1**	**2**	**3**	**4**	**5**	**GA**	GB^*^
The CVLTS helps train veterinary surgeons for total laparoscopic gastropexy (TLG)	0	0	0	3 (18.75%)	13 (81.25%)	4.7	4.9
CVLTS would help me improve my PLG skills and apply them to my patients	0	0	0	4 (25%)	12 (75%)	4.8	4.7
CVLTS is useful for training veterinary medical novices for TLG	0	0	1 (6.25%)	1 (6.25%)	14 (87.5%)	4.7	4.9
Considers it valuable to include CVLTS in laparoscopy training programs for veterinary novices before practicing in the operating room	0	0	0	6 (37.5%)	10 (62.5%)	4.5	4.7
Usefulness for error reduction	0	0	0	5 (31.25%)	11 (68.75%)	4.5	4.8
Usefulness for the evaluation of surgical proficiency for TLG in canines	0	0	1 (6.25%)	6 (37.5%)	9 (56.25%)	4.2	4.7
Utility compared to the use of experimental animals or actual patients	0	0	2 (12.5%)	6 (37.5%)	8 (50%)	4.2	4.5
Visual realism of CVLTS anatomy (it is didactic and sized for canine TLG training)	0	0	3 (18.75%)	6 (37.5%)	7 (43.75%)	3.7^*^	4.6
Realistic image quality and brightness	0	0	1 (6.25%)	6 (37.5%)	9 (56.25%)	4.2	4.7
Realism in the difficulty during TLG in canines	0	0	1 (6.25%)	5 (31.25%)	10 (62.5%)	4.2	4.8
Realism in the consistency of the fabric and materials used	0	1 (6.25%)	0	7 (43.75%)	8 (50%)	3.8	4.7
Realism regarding total laparoscopic gastropexy	0	0	1 (6.25%)	6 (37.5%)	9 (56.25%)	4.2	4.7
How stimulating is this training?	0	0	0	6 (37.5%)	10 (62.5%)	4.5	4.7
The usefulness of training for teaching	0	0	0	1 (6,25%)	15 (93,75%)	5,0	4,9

* Items rated on a Likert-type scale range from 1 to 5. Where “1” represents the lowest level or “strongly disagree” performance, 2 “dissent”, 3 “neutral”, 4 “in agreement”, and 5 ideal performance is considered “strongly agree”. CVLTS, CALMA Veterinary Lap-trainer Simulator; TLG, Pure Laparoscopic Gastropexy; GA, Group A; GB, Group B. ^*^Statistically significant differences between groups (p < 0.05).

### Statistical analysis

Descriptive statistics were calculated. Continuous data were expressed as mean values and ranges, and categorical data were expressed as frequencies for each group. To compare the survey responses and determine significant statistical differences (*p* < 0.05) between the two groups, the Chi-square test was run. The correlation between the two groups ratings was determined with the Spearman test. To compare the age and experience between groups, Student's *t*-test was run. All statistical analyses were performed using R statistical software under the R-Studio platform.

## Results

### Demographic data

The average age of the participants in group A was 40.2 ± 4.7, and 83.33% were right-handed. For group B the average age was 41.2 ± 8.3, and 100% were right-handed (*t* = 0.277, *p* = 0.786). Veterinary thoracoscopy and laparoscopic experience were reported for each group at 11.7 ± 6.6 years for group A and 6.3 ± 7.7 years for group B (*t* = 1.418, *p* = 0.178).

### Visual analog scale

The participants scores were 77.5 ± 24.2 and 46.2 ± 8.6 mm for groups A and B, respectively (*t* = 3.7803, *p* = 0.002).

### Satisfaction survey

The overall mean content validity for the model (sum of all scores) was 4.7/5. The participants “agree” or “strongly agree” with the items in this block. The remaining questions had an average face validity score for the model (sum of all scores) of 4.5/5. This means a degree of satisfaction “agree” or “strongly agree” with what was asked in this block of questions. No significant statistical differences were observed between the responses of the two groups (*p* < 0.05), except for the question “visual realism of CVLTS anatomy,” where statistical differences were observed (*p* = 0.036). When evaluating the association of the test response sets, a correlation coefficient of *r* = 0.6584 (*p* = 0.01046) was determined between groups A and B, indicating that the scores of the two groups are highly correlated. If asked about the usefulness of training veterinary surgeons and veterinary novices in pure laparoscopic gastropexy, 81 and 87% “strongly agreed” on the usefulness of the didactic medium as a training method, respectively. Similarly, 62.5% indicated that they “strongly agreed” that veterinary medical novices should meet this type of simulator before contacting the surgical environment. One participant stated that “he did not doubt the usefulness of the simulator but that the training for novices in TLG was an advanced laparoscopic technique – the technique is complicated for that level of teaching.” For assessing surgical skill acquisition and error correction to perform gastropexy, 75% and 68.75 strongly agreed on the usefulness of developing surgical skills while decreasing errors. When asked about the usefulness of the simulator as a surgical skill assessment tool for pure laparoscopic gastropexy, 93.75% “agreed” or “strongly agreed” that it was a helpful assessment tool for this procedure. One participant commented: “Being a technique that demands high intracorporeal suturing skill makes it difficult to assess it on the simulator.” Finally, 87.5% “agreed” or “strongly agreed” that the didactic medium can be an alternative to experimental animals and the use of actual patients for TLG training.

The concerns expressed by the participants were: “the handling of tissue whether in cadavers or real patients is different,” also “there are additional factors such as bleeding and normal movements of internal organs that are difficult to reproduce.” 93.75% “agreed” or “strongly agreed” that the degree of difficulty offered by the simulator during the simulated TLG exercise was realistic, as was the consistency of the fabric and materials used.

**Figure 3:**
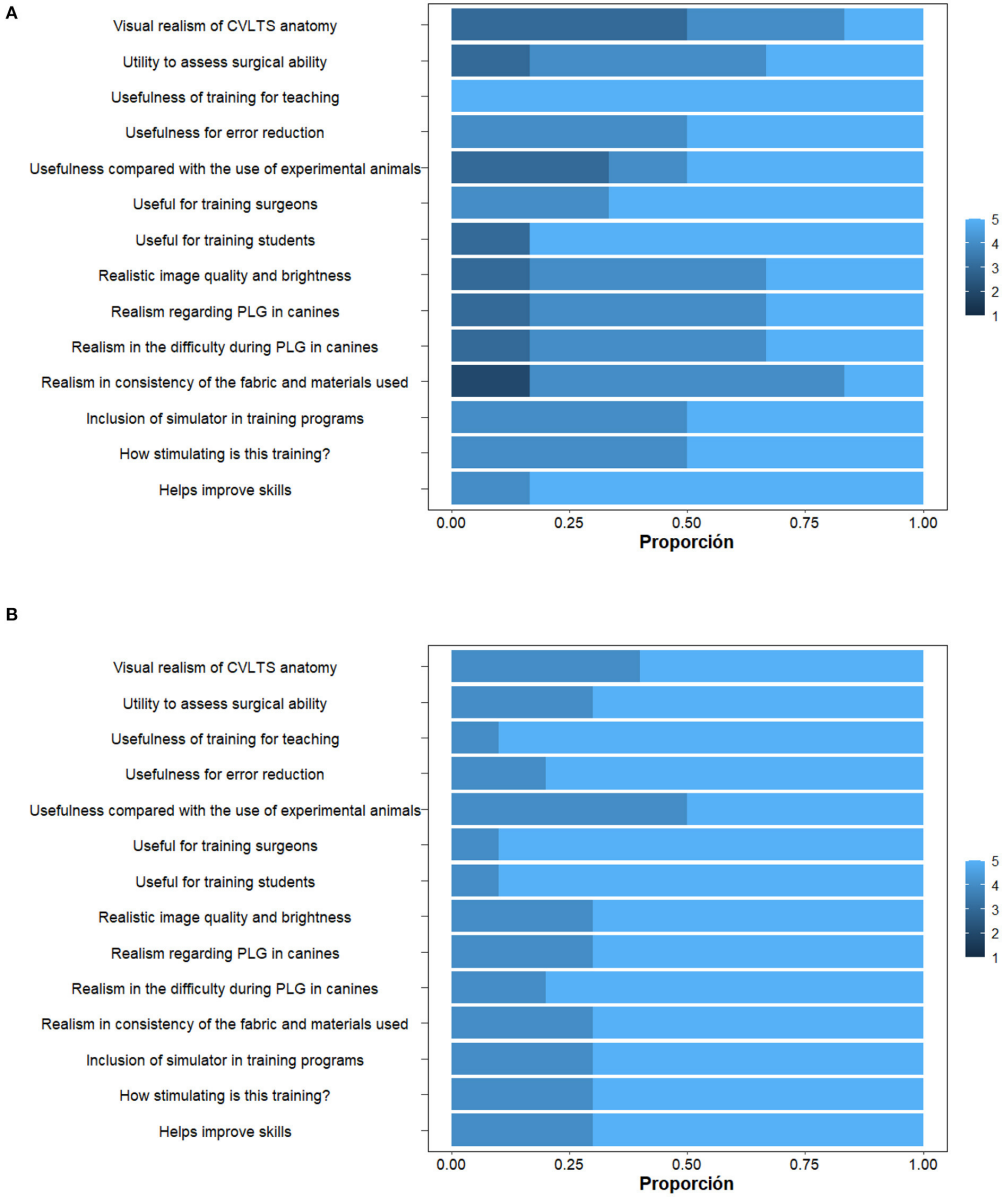
Response distribution graphs between groups. **(A)** Group A. **(B)** Group B.

One participant commented on this item: “the simulator makes the development of the technique more difficult due to the difficulty in moving the left instrument during the suturing phases,” “anatomically, the pig's stomach is different from that of the dog.” 81.25 and 93.75% of the participants “agreed” or “strongly agreed” that the simulator has adequate visual anatomical realism, image quality, and training brightness. When asked about the point realism for TLG in canines, 93.75% indicated “agree” or “strongly agree” with the simulated procedure. Some comments regarding the appearance were: “the port limits the movements of the left hand,” “there is a certain degree of fatigue in the left hand,” and “the image is very clear (very light) concerning the abdominal cavity.” 62% of participants “strongly agreed” that training was stimulating and the usefulness of the training on the simulator for teaching pure laparoscopic gastropexy (Figures 3A,B).

## Discussion

The present work presents the results of the face and content validation of a simulator that is a faithful copy of the ergonomics of the canine abdomen of a giant-sized animal. These findings are based on analyzing two groups of veterinarians with medium and advanced experience in laparoscopic-assisted gastropexy and total laparoscopic gastropexy (TLG). Our research question asks whether possible to develop a simulation model of the abdominal cavity of a canine for the training of advanced laparoscopic techniques in simulation different from traditional box models.

The CVLTS was based on Great Dane patients since this breed reports the highest incidence of presentation of DGV syndrome (6, 25). The highest incidence of GDV syndrome has been reported in the Great Dane breed, with a 42.4% risk (6) and a 10 Odd Ratio (OR) (25). Additionally, the alignment of the ports was considered according to the precise surgical technique along the ventral midline (11) and the patient's position, which is a fundamental piece since it has been proven that it influences training with such simulators. In a study that evaluated whether the essential laparoscopic surgical skill obtained in horizontal training would be transferred to the vertical plane and vice versa, it was found that this did not happen. Therefore, the development of laparoscopic training models where surgical skills obtained in each plane are developed is recommended (24).

Considering the degree of complexity involved in TLG because it requires excellent intracorporeal suturing skills because of the longer execution time compared to the assisted technique (6, 11), and intraoperative complications such as gastric perforation (3), we believe it is essential that these points should be trained in a safe, simulated environment, where repetitions can be performed with feedback on the different surgical skills necessary to reverse these items. Endoscopy- (4) and laparoscopy- (5) assisted gastropexy and TLG with intracorporeal suturing (6, 7) are considered among the prophylactic minimally invasive surgical techniques reported to prevent DGV syndrome.

Although it has been proven that the laparoscopic-assisted gastropexy technique can be performed in a shorter time (28 min, range, 20–41 min), it has a more significant impact on postoperative activity, especially in dogs submitted to TLG heavier than 30 kg (6) and positively impacts postoperative complications such as inflammation and infection around the gastropexy site (12, 26). In agreement with the findings of different investigations, the training of pure laparoscopic gastropexy with the intracorporeal suture is justified by a lower reduction in postoperative activity and fewer complications (6, 12) concerning patients who undergo the laparoscopic-assisted gastropexy technique that requires an incision through all the layers of the abdominal wall musculature. The statistical difference found in the responses on the “visual realism of the CVLTS anatomy” was due to the discomfort of the left hand and the anatomical differences of the anatomical model used for the exercises reported by a participant of the expert group. TLG with the intracorporeal suture is considered a highly complex surgical technique, mainly from the ergonomic point of view.

For this reason, it has been recommended that the three linear ports be located slightly on the right hemiabdomen (12), especially the most caudal port (suprapubic), which allows for better triangulation (11). Our simulator included a wider silicone port for the left-hand port to increase the range of motion of that limb, but it was not enough. Therefore, a larger and less rigid silicone port will be placed in the access point of the left hand (left portal) that allows a more excellent range of movements when performing the intracorporeal suture. This would improve ergonomics during exercises.

We include the pig stomach in our composite model because of its low cost and ease of procurement and technically because it perfectly simulates the layers of the dog's stomach that in our training had to be incised and sutured. Although LGT has been described without incision of the seromuscular layer of the stomach (12), for the didactic purpose of the simulator it is necessary to apply the basic cutting skill that is necessary for the same work mentioned in the seromuscular layer of the abdominal wall. Observations concerning model realism, such as motion hemodynamic (bleeding) simulation, will always be a challenge for the creation and study of medical simulation in surgery (17), but we believe that it is dependent on what you are looking to train.

Validity equals the degree to which evidence and theory support interpretations of test scores for the intended uses of the tests. Validation currently refers to the validation process that involves accumulating relevant evidence to provide a solid scientific basis for the proposed scoring interpretations than to the different classical validity types (27). This has been demonstrated in systematic reviews that show the trend in human medicine research on the assessment of technical skills who define and collect sources of validity evidence using formal validity frameworks such as Messick's modern validity framework (20, 28, 29) which consists of 5 different sources of validity evidence: content, response process, internal structure, relationships with other variables and consequences of the evaluation/test (20). Sanchez-Hurtado et al. proposed a methodology for the validation of surgical simulators in laparoscopic surgery from design and development to data recording and interpretation, which includes internal validity (fidelity, verification, calibration, and reliability) and external validity (subjective and objective validation strategies) (18). Levels comprising the classic validity framework include (i) Content validity, ensuring that all relevant dimensions are measured within what is to be trained or evaluated (ii) Construct validity, which is indispensable for detecting differences between groups exhibiting distinct levels of competence (iii) Concurrent validity, where the correlation between the test results and the validated simulator criteria is evaluated (iv) Predictive validity, which is the ability to predict future performance in an actual environment or experimental model. Finally, (v) Face validity, which measures the degree of realism of the simulator (19). We are aware that subjective validation strategies (content and apparent validation) are currently considered outdated. Our simulator was conceived under this modified proposal of the classic validation framework, where international experts were included in the internal validation, then began the process of face and content validation.

Although the participants included in the study had varying degrees of experience in veterinary laparoscopic surgery, for the most part, they indicated that the model would be helpful for basic skills training to perform TLG. The R-value supports this finding, indicating a correlation between the ratings of the two groups, and the chi-square test indicated that there is no difference in the ratings for the two groups, indicating that the variance between the ratings of the two groups (A and B) are correlated and not statistically different. Although there are no specific rates for subjective validity, previously evaluated models such as the high-fidelity simulated laparoscopic ovariectomy (SLO) model obtained an overall face validity measure of 64.2/100 (17). Similarly, French et al. validated a laparoscopic abdominal simulator to prepare for laparoscopic ovariectomy in live dogs. They found that participants “agreed” or “strongly agreed” that the simulator was easy to use and adequately realistic for practice and evaluation of laparoscopic ovariectomy, even before performing the procedure on a living patient (30). Overall, the acceptance of this type of simulated training media for veterinarians is acceptable to the participants (15), most likely due to the lack of efficient and safe methods for training basic and advanced skills in veterinary medicine. At present, we only have simulators such as Simulvet^®^ [Centro de cirugía de mínima invasión Jesús Usón (CCMIJU), Cáceres, Spain] (15) that allow the acquisition of basic skills and the Mayo Endoscopy Simulated Image (MESI, Sawbones, Pacific Research Laboratories Inc, Vashon, WA) canine abdominal model, which was validated for a specific surgical technique in laparoscopic spaying (17) conceived with the size and space limitations of our patients in mind.

In addition, the design of the simulators must allow an exciting environment that motivates the student to improve their surgical skills, with perfectly defined tasks and with timely feedback that does not allow errors to be fixed in the Student and that leads to the fixation of surgical skill (31). Simulation offers a safe environment in which psychomotor skills can be increased in a controlled and more efficient, and time-effective manner to train surgeons without posing a risk to patients or trainees (32). In general, our proposal essentially covers what is proposed in the scientific literature in terms of the training model that should be followed at present to teach laparoscopic techniques in veterinary medicine.

The difficulty in performing intracorporeal suturing in laparoscopic surgery is often due to visual limitations and mechanical difficulties that can be worked on even before acquiring basic laparoscopic skills since it is not a prerequisite for training in intracorporeal suturing (13). Likewise, isolated training in a simulated environment without true automation of performing intracorporeal suturing in the applied environment is inadequate (14). Therefore, we seek to increase vet surgeons' exposure to this skill in a context closer to the real one of laparoscopic suturing in patients.

In conclusion, preliminarily, the CVLTS and its training program showed good acceptance in terms of realism and content by the group of veterinarians who performed the tasks to perform a simulated TGL. Additionally, it is considered a valuable tool for teaching intracorporeal suturing and technique with an acceptable stimulating factor for training. However, there were limitations, such as the small number of expert participants to compare evaluations with other simulators to determine the concurrent validity of the simulator. It also includes the determination of construct and predictive validity of the training program and CVLTS with a group of novices. For this reason, further research studies on teaching and learning methods in veterinary laparoscopic surgery are needed to determine the usefulness of the model for pre-training before engaging in the actual surgical environment.

## Study limitations

In this preliminary study, only one testing session was conducted for the participants. Surgical performance was not evaluated through objective evaluations such as Objective structured assessment of technical skill (OSATS) and/or Global Operative Assessment of Laparoscopic Skills (GOALS) or sensitive performance metrics such as execution time, the economy of movement, smoothness of the movement, angle of trajectory, among others. therefore, the CVLTS was not tested in a teaching or educational setting. This simulator does not allow abdominal access training for port placement and does not simulate abdominal movement and bleeding. Preliminarily, only the subjective strategies were applied for content and apparent validation.

## Data availability statement

The raw data supporting the conclusions of this article will be made available by the authors, without undue reservation.

## Ethics statement

The studies involving human participants were reviewed and approved by the Comité de Bioética de Investigación en Humanos CBE-SIU. The patients/participants provided their written informed consent to participate in this study.

## Author contributions

CO-P, JM-E, and JL designed the study and developed the surveys. CO-P monitored the procedures. CO-P, JM-E, JL, GGM, and CR-B analyzed and interpreted the data and drafted the manuscript. All authors read and approved the final manuscript.

## Funding

This work was funded by grant # 727-2015 from the Ministerio de Ciencias y Tecnología de Colombia (Minciencias) awarded to CO-P, the University of Antioquia through grants to OHVRI and Biotechnology groups (Estrategia de Sostenibilidad de grupos 2021–2022) and University of Cordoba through Doctoral studies scholarship to CO-P.

## Conflict of interest

The authors declare that the research was conducted in the absence of any commercial or financial relationships that could be construed as a potential conflict of interest.

## Publisher's note

All claims expressed in this article are solely those of the authors and do not necessarily represent those of their affiliated organizations, or those of the publisher, the editors and the reviewers. Any product that may be evaluated in this article, or claim that may be made by its manufacturer, is not guaranteed or endorsed by the publisher.
